# Supplementary material for: Using Genomics Feature Selection Method in Radiomics Pipeline Improves Prognostication Performance in Locally Advanced Esophageal Squamous Cell Carcinoma—A Pilot Study
Source: Cancers (Basel). 2021 Apr 29;13(9):2145. doi: 10.3390/cancers13092145 (PMC8124289; doi:10.3390/cancers13092145)
Supplement: Supplementary file 1 [file cancers-13-02145-s001.zip › cancers-1141716 supplementary.pdf]

Using Genomics Feature Selection Method in Radiomics Pipeline Improves Prognostication Performance in Locally Advanced Esophageal Squamous Cell Carcinoma—A Pilot Study

Chen-Yi Xie, Yi-Huai Hu, Joshua Wing-Kei Ho, Lu-Jun Han, Hong Yang, Jing Wen, Ka-On Lam, Ian Yu-Hong Wong, Simon Ying-Kit Law, Keith Wan-Hang Chiu, Jian-Hua Fu and Varut Vardhanabhuti

Supplementary Method. CT acquisition, pathologic analysis, radiomics feature definition**,** statistical analysis
Supplementary Table S1. The top 10 enriched gene set expression patterns by G:profiler
Supplementary Table S2. The differentially expressed genes analyzed by limma and overlapped genes for genomics feature selection for correlation analysis with radiomics features.
Supplementary Table S3. Numbers of selected features for model constructions
Supplementary Table S4. Description of selected radiomic features in the radiomics models for the construction of nomogram
Supplementary Figure S1. Flowchart of patient selection
Supplementary Figure S2. Correlation between selected radiomics features and the overlapped genes.
Supplementary Figure S3. Calibration plot of 3-year and 5-year time-dependent ROC curves in the training, internal test and external test set.
Supplementary Figure S4. Decision curve analysis of nomograms
Supplementary Figure S5. Overall survival for patients from high-risk and low-risk groups stratified by nomogram predictions.
Supplementary Figure S6. Survival for patients from high-risk and low-risk groups stratified by delta radiological features.

Supplementary Method

1. CT acquisition

Parameter	Institution 1	Institution 2
Scanner	Aquilion TSX-101A (Toshiba Medical Systems, Tokyo, JP) or Discovery 750 HD (GE Healthcare, Milwaukee, WI)	Discovery VCT, GE Healthcare (GE Healthcare, Milwaukee, WI)
Dose of iodinated CT contrast agent	1.5 ml/kg	100 mL
Injection rate of iodinated CT contrast agent	2.5 ml/s	3 ml/s
Slice thickness (mm)	2.5 (median)	5 (median)
Tube voltage (kVp)	120	120
Tube current (mA)	200-400	200-300

2. Pathologic Analysis

Pathologic complete response (pCR) was defined as no evidence of residual tumor cells in the primary site and resected lymph nodes of the operative specimens. For institution 1, reports on pathologic examination contained the tumor type and extension, proximal and distal resection margins, tumor regression grade, and lymph node status. For institution 2, pathological report also described the circumferential resection.

3.Radiomics Feature definitions

Handcrafted radiomics features were computed from the radiologist-drawn ROIs using an open-source python package PyRadiomics [1]. Detailed calculations of handcrafted radiomics features are described and provided in online documentation of PyRadiomics (https://pyradiomics.readthedocs.io/en/latest/features.html). The resampled voxel sizes were set to $1\times 1 \times 5$ mm^3 voxels to standardize the slice thickness. Image intensities were binned by 25 HU and voxel array shift were set on 1000. Segmented voxels were resampled at the range of 50 to 400 HU including the whole tumor and excluding the air and bone tissues. Defined radiomic image features without/after wavelet filtration that described tumor characteristics were extracted. Wavelet filtration filtered original image directionally with x, y and z directions respectively (H: High pass filter, L: Low pass filter), yielding 8 different combinations of decompositions. The extracted radiomics features can be divided into 3 groups: (I) first-order statistics, (II) shape features, and (III) second-order features. Most features defined below were in accord with feature definitions as described by the Imaging Biomarker Standardization Initiative (IBSI), which were available in a separate document by Zwanenburg et al.[2].

4.1. First Order Features

First-order statistics describe the distribution of voxel intensities within the image region defined by the mask through commonly used and basic metrics.

Let:

- $\mathbf{X}$ be a set of Np voxels included in the ROI
- $\mathbf{P}(i)$ be the first order histogram with Ng discrete intensity levels, where Ng is the number of non-zero bins, equally spaced from 0 with a width defined in the bin width parameter
- $p(i)$ be the normalized first-order histogram and equal to $\mathbf{P}(i)/Np$

1) Median

The median gray level intensity within the ROI.

2). Skewness

$$skewness = \frac{\mu_3}{\sigma^3} = \frac{\frac{1}{N_p}\sum_{i=1}^{N_p}(\mathbf{X}(i) - \bar{X})^3}{\left(\sqrt{\frac{1}{N_p}\sum_{i=1}^{N_p}(\mathbf{X}(i) - \bar{X})^2}\right)^3}$$

Skewness measures the asymmetry of the distribution of values about the Mean value. Depending on where the tail is elongated and the mass of the distribution is concentrated, this value can be positive or negative.

4.2 Second-Order Features

A. Gray Level Co-occurrence Matrix (GLCM) Features

A Gray Level Co-occurrence Matrix (GLCM) [3] of size $Ng \times Ng$ describes the second-order joint probability function of an image region constrained by the mask and is defined as $\mathbf{P}(i,j|\delta,\theta)$. The $(i,j)^{th}$ element of this matrix represents the number of times the combination of levels i and j occur in two pixels in the image, that are separated by a distance of δ pixels along angle θ. The distance δ from the center voxel is defined as the distance according to the infinity norm. For δ=1, this results in 2 neighbors for each of 13 angles in 3D (26-connectivity) and for δ=2 a 98-connectivity (49 unique angles).

As a two dimensional example, let the following matrix **I** represent a 5x5 image, having 5 discrete grey levels:

$$\mathbf{I} = \begin{bmatrix} 1 & 2 & 5 & 2 & 3 \\ 3 & 2 & 1 & 3 & 1 \\ 1 & 3 & 5 & 5 & 2 \\ 1 & 1 & 1 & 1 & 2 \\ 1 & 2 & 4 & 3 & 5 \end{bmatrix}$$

For distance δ=1 (considering pixels with a distance of 1 pixel from each other) and angle θ=0° (horizontal plane, i.e. voxels to the left and right of the center voxel), the following symmetrical GLCM is obtained:

$$\mathbf{P} = \begin{bmatrix} 6 & 4 & 3 & 0 & 0 \\ 4 & 0 & 2 & 1 & 3 \\ 3 & 2 & 0 & 1 & 2 \\ 0 & 1 & 1 & 0 & 0 \\ 0 & 3 & 2 & 0 & 2 \end{bmatrix}$$

Let:

- ϵ be an arbitrarily small positive number ($\approx 2.2\times10^{-16}$)
- $\mathbf{P}(i,j)$ be the co-occurence matrix for an arbitrary δ and θ
- $p(i,j)$ be the normalized co-occurence matrix and equal to $\mathbf{P}(i,j)\sum\mathbf{P}(i,j)$
- Ng be the number of discrete intensity levels in the image
- $px(i)=\sum Ngj=1P(i,j)$ be the marginal row probabilities
- $py(j)=\sum Ngi=1P(i,j)$ be the marginal column probabilities
- σx be the standard deviation of px
- σy be the standard deviation of py

By default, the value of a feature is calculated on the GLCM for each angle separately, after which the mean of these values is returned.

1). Cluster Shade

$$cluster\ shade = \sum_{i=1}^{N_g}\sum_{j=1}^{N_g}\left(i + j - \mu_x - \mu_y\right)^3 p(i,j)$$

Cluster Shade is a measure of the skewness and uniformity of the GLCM. A higher cluster shade implies greater asymmetry about the mean.

2). Correlation

$$correlation = \frac{\sum_{i=1}^{N_g} \sum_{j=1}^{N_g} p(i,j)ij - \mu_x \mu_y}{\sigma_x(i)\sigma_y(j)}$$

Correlation is a value between 0 (uncorrelated) and 1 (perfectly correlated) showing the linear dependency of gray level values to their respective voxels in the GLCM.

3). Joint Average

$$joint\ average = \mu_x = \sum_{i=1}^{N_g} \sum_{j=1}^{N_g} p(i,j)i$$

Returns the mean gray level intensity of the i distribution.

B. Gray Level Dependence Matrix (GLDM) Features

A Gray Level Dependence Matrix (GLDM) quantifies gray level dependencies in an image.[4] A gray level dependency is defined as the number of connected voxels within distance δ that are dependent on the center voxel. A neighboring voxel with gray level jj is considered dependent on center voxel with gray level i if $|i-j|\le\alpha$. In a gray level dependence matrix $\mathbf{P}(i,j)$ the $(i,j)^{th}$ element describes the number of times a voxel with gray level i with j dependent voxels in its neighborhood appears in image. As a two-dimensional example, consider the following 5x5 image, with 5 discrete gray levels:

$$\mathbf{I} = \begin{bmatrix} 5 & 2 & 5 & 4 & 4 \\ 3 & 3 & 3 & 1 & 3 \\ 2 & 1 & 1 & 1 & 3 \\ 4 & 2 & 2 & 2 & 3 \\ 3 & 5 & 3 & 3 & 2 \end{bmatrix}$$

For α=0 and δ=1, the GLDM then becomes:

$$\mathbf{P} = \begin{bmatrix} 0 & 1 & 2 & 1 \\ 1 & 2 & 3 & 0 \\ 1 & 4 & 4 & 0 \\ 1 & 2 & 0 & 0 \\ 3 & 0 & 0 & 0 \end{bmatrix}$$

Let:

- Ng be the number of discreet intensity values in the image
- Nd be the number of discreet dependency sizes in the image
- Nz be the number of dependency zones in the image, which is equal to $\sum_{i=1}^{N_g} \sum_{j=1}^{N_d} \mathbf{P}(i,j)$
- $\mathbf{P}(i,j)$ be the dependence matrix
- $p(i,j)$ be the normalized dependence matrix, defined as $p(i,j) = \frac{\mathbf{P}(i,j)}{N_z}$

1). Dependence Variance (DV)

$$DV = \sum_{i=1}^{N_g}\sum_{j=1}^{N_d} p(i,j)(j-\mu)^2, \text{ where} \mu = \sum_{i=1}^{N_g}\sum_{j=1}^{N_d} jp(i,j)$$

Measures the variance in dependence size in the image.

2). Dependence Non-Uniformity Normalized (DNN)

$$DNN = \frac{\sum_{j=1}^{N_d}\left(\sum_{i=1}^{N_g} \mathbf{P}(i,j)\right)^2}{N_z^2}$$

Measures the similarity of dependence throughout the image, with a lower value indicating more homogeneity among dependencies in the image. This is the normalized version of the DLN formula.

C. Gray Level Run Length Matrix (GLRLM) Features

A Gray Level Run Length Matrix (GLRLM) quantifies gray level runs, which are defined as the length in number of pixels, of consecutive pixels that have the same gray level value **[5-8]**. In a gray level run length matrix $\mathbf{P}(i,j|\theta)$, the $(i,j)^{th}$ element describes the number of runs with gray level i and length j occur in the image (ROI) along angle θ.

As a two-dimensional example, consider the following 5x5 image, with 5 discrete gray levels:

$$\mathbf{I} = \begin{bmatrix} 5 & 2 & 5 & 4 & 4 \\ 3 & 3 & 3 & 1 & 3 \\ 2 & 1 & 1 & 1 & 3 \\ 4 & 2 & 2 & 2 & 3 \\ 3 & 5 & 3 & 3 & 2 \end{bmatrix}$$

The GLRLM for θ=**0**, where 0 degrees is the horizontal direction, then becomes:

$$\mathbf{P} = \begin{bmatrix} 1 & 0 & 1 & 0 & 0 \\ 3 & 0 & 1 & 0 & 0 \\ 4 & 1 & 1 & 0 & 0 \\ 1 & 1 & 0 & 0 & 0 \\ 3 & 0 & 0 & 0 & 0 \end{bmatrix}$$

Let:

- Ng be the number of discreet intensity values in the image
- Nr be the number of discreet run lengths in the image
- Np be the number of voxels in the image
- $Nr(\theta)$ be the number of runs in the image along angle θ, which is equal to $\sum_{i=1}^{N_g}\sum_{j=1}^{N_r} \mathbf{P}(i,j|\theta)$ and $1 \le Nr(\theta) \le Np$
- $\mathbf{P}(i,j|\theta)$ be the run length matrix for an arbitrary direction θ
- $p(i,j|\theta)$ be the normalized run length matrix, defined as $p(i,j|\theta) = \frac{\mathbf{P}(i,j|\theta)}{N_r(\theta)}$

By default, the value of a feature is calculated on the GLRLM for each angle separately, after which the mean of these values is returned. If distance weighting is enabled, GLRLMs are weighted by the distance between neighboring voxels and then summed and normalized. Features are then calculated on the resultant matrix. The distance between neighboring voxels is calculated for each angle using the norm specified in 'weightingNorm'.

1). Long Run High Gray Level Emphasis (LRHGLE)

$$LRHGLRE = \frac{\sum_{i=1}^{N_g}\sum_{j=1}^{N_r}\mathbf{P}(i,j|\theta)i^2j^2}{N_r(\theta)}$$

LRHGLRE measures the joint distribution of long run lengths with higher gray-level values.

2). Size-Zone Non-Uniformity Normalized (SZNN)

$$SZNN = \frac{\sum_{j=1}^{N_s}\left(\sum_{i=1}^{N_g}\mathbf{P}(i,j)\right)^2}{N_z^2}$$

SZNN measures the variability of size zone volumes throughout the image, with a lower value indicating more homogeneity among zone size volumes in the image. This is the normalized version of the SZN formula.

D. Gray Level Size Zone Matrix (GLSZM) Features

A Gray Level Size Zone (GLSZM) quantifies gray level zones in an image. A gray level zone is defined as the number of connected voxels that share the same gray level intensity. A voxel is considered connected if the distance is 1 according to the infinity norm (26-connected region in a 3D, 8-connected region in 2D). In a gray level size zone matrix $P(i,j)$ the $(i,j)^{th}$ element equals the number of zones with gray level i and size j appear in image. Contrary to GLCM and GLRLM, the GLSZM is rotation independent, with only one matrix calculated for all directions in the ROI [9]. As a two-dimensional example, consider the following 5x5 image, with 5 discrete gray levels:

$$\mathbf{I} = \begin{bmatrix} 5 & 2 & 5 & 4 & 4 \\ 3 & 3 & 3 & 1 & 3 \\ 2 & 1 & 1 & 1 & 3 \\ 4 & 2 & 2 & 2 & 3 \\ 3 & 5 & 3 & 3 & 2 \end{bmatrix}$$

The GLSZM then becomes:

$$\mathbf{P} = \begin{bmatrix} 0 & 0 & 0 & 1 & 0 \\ 1 & 0 & 0 & 0 & 1 \\ 1 & 0 & 1 & 0 & 1 \\ 1 & 1 & 0 & 0 & 0 \\ 3 & 0 & 0 & 0 & 0 \end{bmatrix}$$

Let:

- Ng be the number of discreet intensity values in the image
- Ns be the number of discreet zone sizes in the image
- Np be the number of voxels in the image
- Nz be the number of zones in the ROI, which is equal to $\sum_{i=1}^{N_g}\sum_{j=1}^{N_s}\mathbf{P}(i,j)$ and $1 \le Nz \le Np$
- $\mathbf{P}(i,j)$ be the size zone matrix
- $p(i,j)$ be the normalized size zone matrix, defined as $p(i,j) = \frac{\mathbf{P}(i,j)}{N_z}$

1). Gray Level Variance (GLV)

$$GLV = \sum_{i=1}^{N_g} \sum_{j=1}^{N_s} p(i,j)(i-\mu)^2$$

GLV measures the variance in gray level intensities for the zones.

E. Neighboring Gray Tone Difference Matrix (NGTDM) Features

A Neighboring Gray Tone Difference Matrix quantifies the difference between a gray value and the average gray value of its neighbors within distance δ. The sum of absolute differences for gray level i is stored in the matrix. Let $\mathbf{X}_{gl}$ be a set of segmented voxels and $x_{gl}(j_x,j_y,j_z) \in \mathbf{X}_{gl}$ be the gray level of a voxel at position (j_x,j_y,j_z).

1). Strength

Strength is a measure of the primitives in an image. Its value is high when the primitives are easily defined and visible, i.e. an image with slow change in intensity but more large coarse differences in gray level intensities.

3.Statistical analysis

A two-tailed P value less than 0.05 was defined as statistically significant. The P values for differences in the clinical characteristics between cohorts were calculated by Fisher exact test or Chi-square test for categorical data, and by Kruskal-Wallis test for numeric data. Radiomics features were harmonized to reduce the multicenter effect caused by different scanner and protocol settings. According to the statistical distribution of the dataset, nonparametric form of the model was adopted in which ComBat determined the transformation for each feature separately using "sva" R package [10]. Feature robustness was tested by intraclass correlation coefficients (ICCs) using "irr" R package [11]. Discrimination ability was assessed by Harrell's concordance indices (C-index) using "Hmisc" R package [12]. The raw genomic data was preprocessed (background correction, log2-transformation and quantile normalization) using the Bioconductor package "affy" [13]. The "limma" package was applied to detect differentially expressed genes (DEGs) between patients with different survival outcome [14]. Cox regression, nomogram construction, and calibration were analyzed by "rms" package [15]. The time-dependent ROC curve analysis were generated using "timeROC" package [16]. The "survival" [17] package was used for survival analysis and graphics.

Supplementary Table S1. The top 10 enriched gene set expression patterns by G:profiler

Term name	Term ID
peptidyl-threonine phosphorylation	GO:0018107
peptidyl-threonine modification	GO:0018210
regulation of peptidyl-threonine phosphorylation	GO:0010799
positive regulation of peptidyl-serine phosphorylation	GO:0033138
positive regulation of ruffle assembly	GO:1900029
negative regulation of ERBB signaling pathway	GO:1901185
positive regulation of protein kinase activity	GO:0045860
positive regulation of otic vesicle morphogenesis	GO:1904120
negative regulation of epidermal growth factor receptor signaling pathway	GO:0042059
regulation of epidermal growth factor receptor signaling pathway	GO:0042058

Supplementary Table S2. The differentially expressed genes analyzed by Limma and overlapped genes for genomics feature selection for correlation analysis with radiomics features.

Differentially expressed genes	Overlapped genes for genomics feature selection
KLK8, STOX1, SPRY2, GPRC5A, LINC02549, IGSF10, MED12L, SKIDA1, COBL, SNRK, NR3C2, ITPK1-AS1, TXNIP, ANKRD26P3, EPS8, GPX3, APCDD1L-DT, KLK6, M1AP, LINC00606, ZNF483, MIR31HG, LINC01904, KIF26B, TUBA3FP, IPW, PWARSN, SGCB, LOC101928196, CABCOCO1, FGF4, TIGD4, PNLIPRP3, LOC105370475, PRCP, CYP27C1, CCDC190	KLK8, STOX1, SPRY2, GPRC5A, IGSF10, COBL, TXNIP, EPS8, GPX3, KLK6, M1AP, ZNF483, KIF26B, SGCB, FGF4, PRCP

Supplementary Table S3. Numbers of selected features for model constructions

Feature selection	Nomogram 1 (with genomics feature selection)	Nomogram 2 (without genomics feature selection)
Initial	2553	2553
Inter-observer variability assessment	2336	2336
Genomics feature selection	1422	Skip
Data-driven machine learning feature selection	8	8

Supplementary Table S4. Description of selected radiomic features in the radiomics models for the construction of nomogram.

Feature index	Category	Stage	Filter	Type	Feature name
Feature 1	Nomogram 1	Pre-nCRT	Wavelet (LLH)	GLCM	Cluster Shade
Feature 2		Pre-nCRT	Wavelet (HLL)	GLDM	Dependence Variance
Feature 3		Pre-nCRT	Wavelet (HLH)	GLRLM	Long Run High Gray Level Emphasis
Feature 4		Post-nCRT	Wavelet (LLH)	First order	Mean
Feature 5		Post-nCRT	Wavelet (HHL)	First order	Mean
Feature 6		Delta	Wavelet (HHL)	GLDM	Dependence Variance
Feature 7	Nomogram 1 & Nomogram 2	Post-nCRT	Wavelet (HLH)	First order	Skewness
Feature 8		Delta	Wavelet (LLL)	GLCM	Correlation
Feature 9	Nomogram 2	Pre-nCRT	Wavelet (LLH)	First order	Mean
Feature 10		Pre-nCRT	Wavelet (HLL)	GLDM	Dependence Non-Uniformity Normaliz[illegible]
Feature 11		Pre-nCRT	Wavelet (HLL)	NGTDM	Strength
Feature 12		Pre-nCRT	Wavelet (HLH)	GLCM	Joint Average
Feature 13		Pre-nCRT	Wavelet (HLH)	GLSZM	Size-Zone Non-Uniformity Normalize[illegible]
Feature 14		Post-nCRT	Wavelet (LLH)	GLSZM	Gray Level Variance

* feature 7 and feature 8 were features that were selected for the construction of Rad-score in both nomogram 1 and nomogram 2

GLCM, Gray Level Co-occurrence Matrix, GLDM, Gray Level Dependence Matrix, GLRLM, Gray Level Run Length Matrix, NGTDM, Neighbouring Gray Tone Difference Matrix, GLSZM, Gray Level Size Zone Matrix

Supplementary Figure S1. Flowchart of patient selection

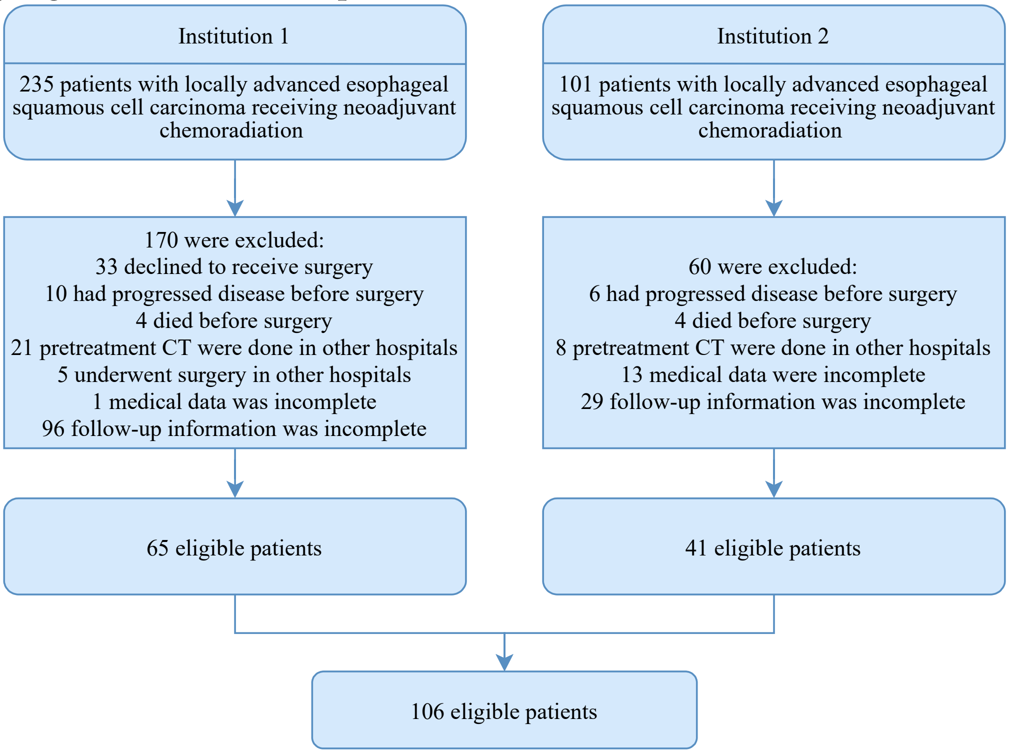

Supplementary Figure S2. Correlation between selected radiomics features and the overlapped genes. Radiomics features in nomogram 1 were derived from genomics feature selection (Radiomics feature 1 to 8). Radiomics features in nomogram 2 without genomics feature selection (Radiomics feature 7 to 14). Feature names were listed in Table 2.

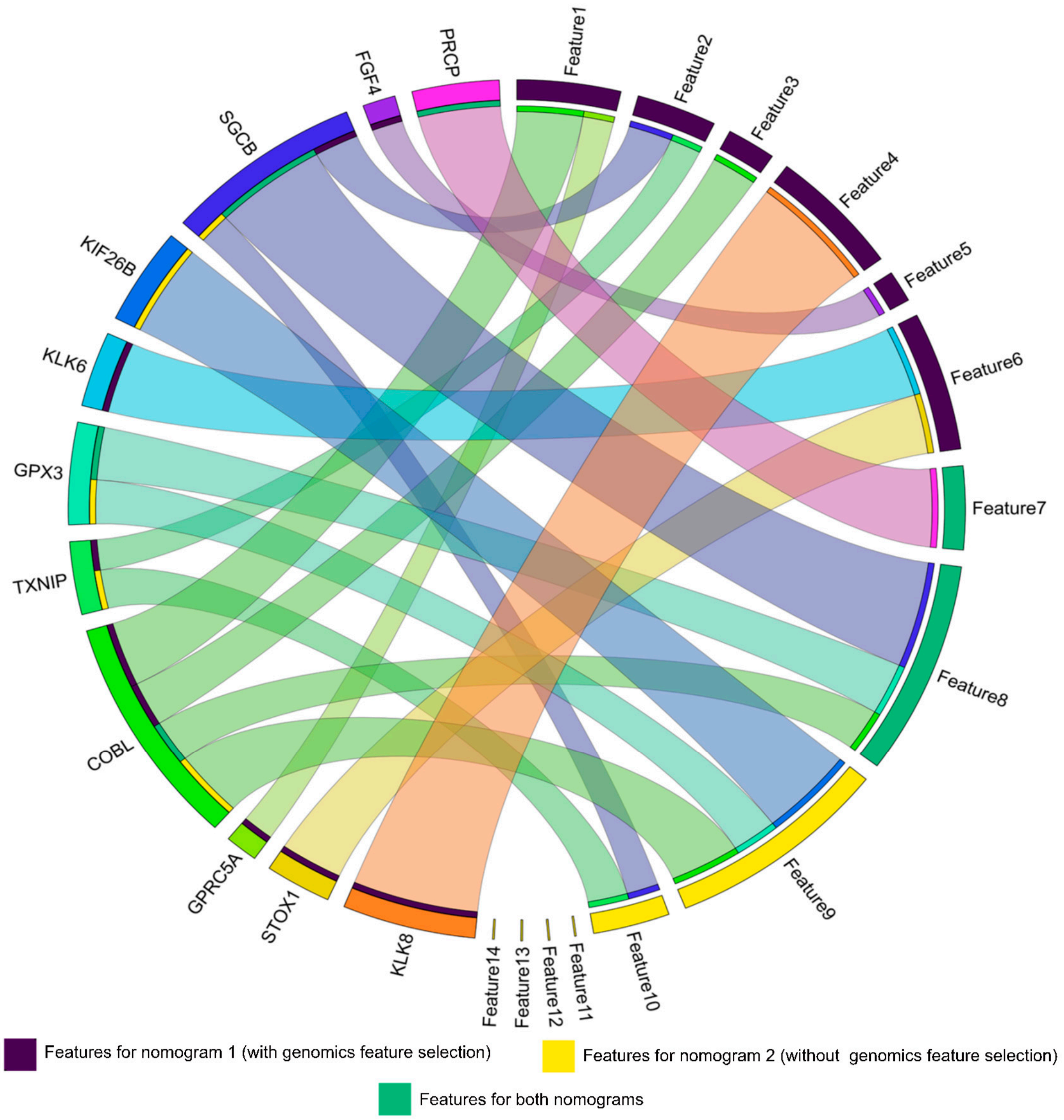

Supplementary Figure S3. Calibration plot of 3-year and 5-year time-dependent ROC curves in the training, internal test and external test set. (A, C, E) nomogram 1 with genomics feature selection. (B, D, F) nomogram 2 without genomics feature selection. ROC = receiver operating characteristic

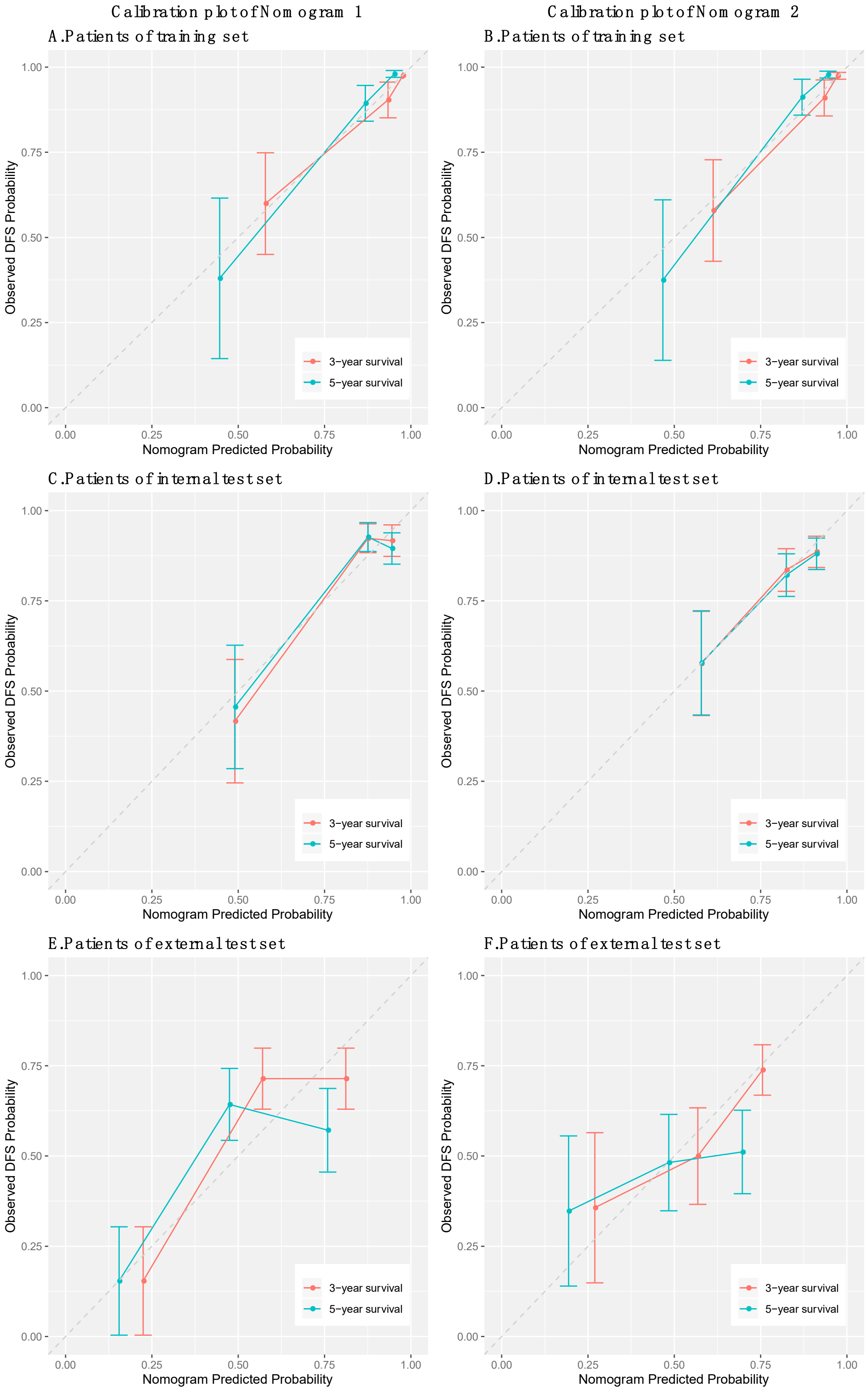

Supplementary Figure S4. Decision curve analysis of nomograms

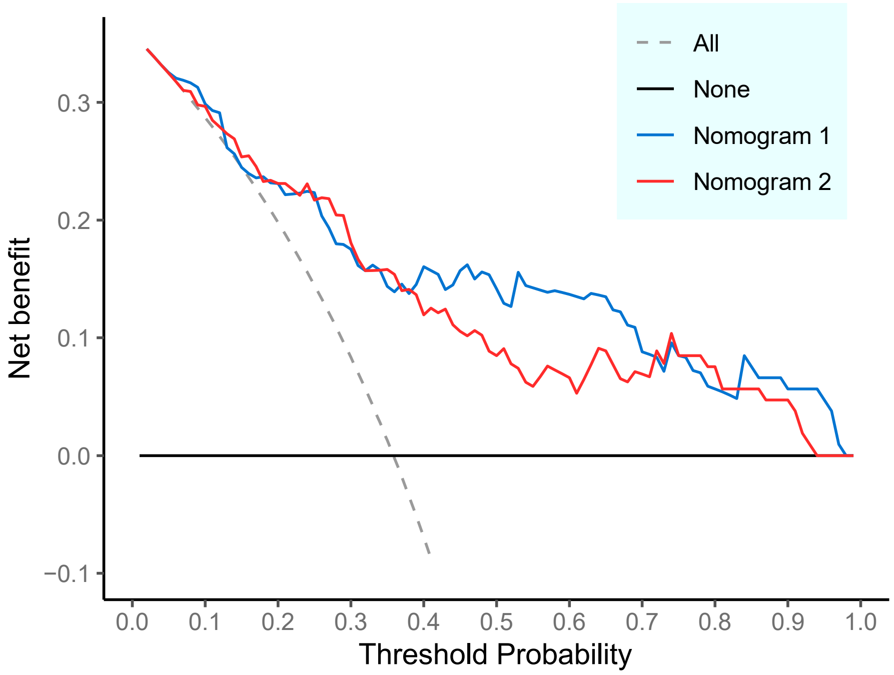

Supplementary Figure S5. Overall survival for patients from high-risk and low-risk groups stratified by nomogram predictions. (A, C, E) nomogram 1 with genomics feature selection. (B, D, F) nomogram 2 without genomics feature selection. Kaplan-Meier curves showing disease-free survival in patients stratified by nomogram predictions in the training, internal test and external test set. The difference between the two curves was compared by the log-rank test.

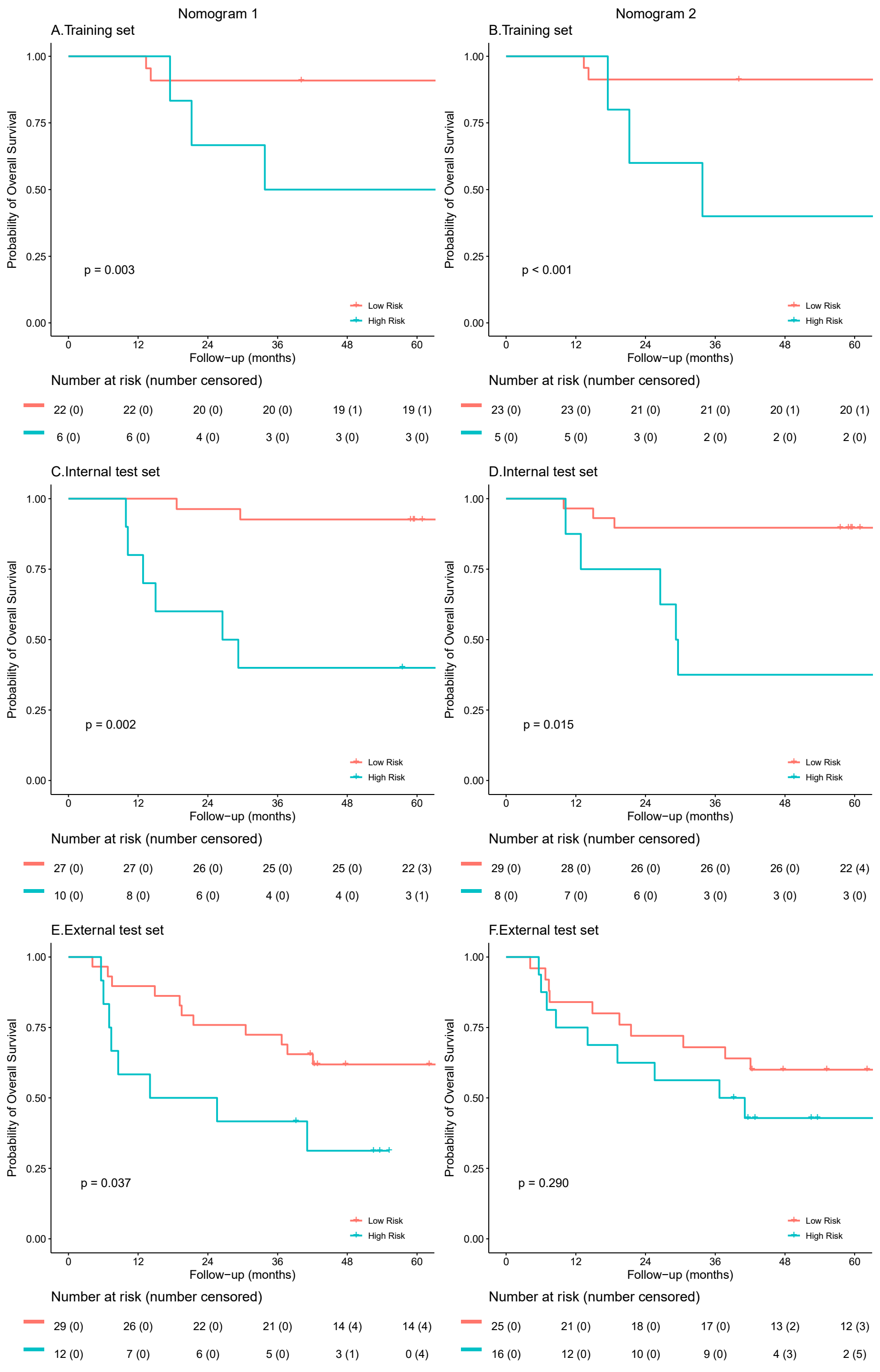

Supplementary Figure S6. Survival for patients from high-risk and low-risk groups stratified by delta radiological features. (A) Delta volume and (B) the delta radiomics feature for the prediction of disease-free survival; (C) Delta volume and (D) the delta radiomics feature for the prediction of overall survival.

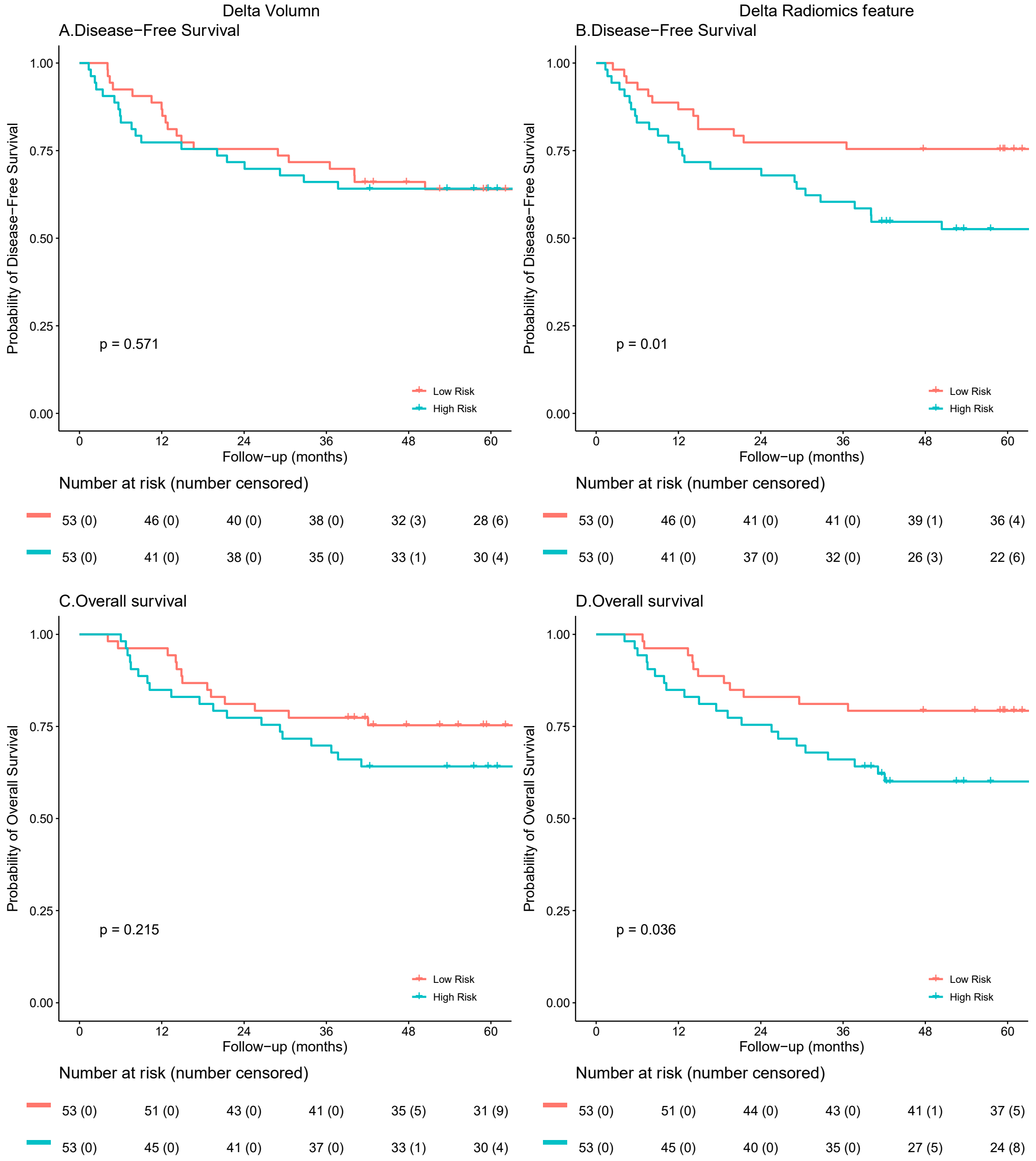

Delta Volumn
Delta Radiomics feature
A.Disease−Free Survival
B.Disease−Free Survival
C.Overall survival
D.Overall survival
Probability of Disease−Free Survival
Probability of Overall Survival
1.00
0.75
0.50
0.25
0.00
0 12 24 36 48 60
Follow−up (months)
p = 0.571
p = 0.01
p = 0.215
p = 0.036
Low Risk
High Risk
Number at risk (number censored)
A: Low Risk: 53 (0) 46 (0) 40 (0) 38 (0) 32 (3) 28 (6)
A: High Risk: 53 (0) 41 (0) 38 (0) 35 (0) 33 (1) 30 (4)
B: Low Risk: 53 (0) 46 (0) 41 (0) 41 (0) 39 (1) 36 (4)
B: High Risk: 53 (0) 41 (0) 37 (0) 32 (0) 26 (3) 22 (6)
C: Low Risk: 53 (0) 51 (0) 43 (0) 41 (0) 35 (5) 31 (9)
C: High Risk: 53 (0) 45 (0) 41 (0) 37 (0) 33 (1) 30 (4)
D: Low Risk: 53 (0) 51 (0) 44 (0) 43 (0) 41 (1) 37 (5)
D: High Risk: 53 (0) 45 (0) 40 (0) 35 (0) 27 (5) 24 (8)

Reference

1. Van Griethuysen, J.J.; Fedorov, A.; Parmar, C.; Hosny, A.; Aucoin, N.; Narayan, V.; Beets-Tan, R.G.; Fillion-Robin, J.-C.; Pieper, S.; Aerts, H.J. Computational radiomics system to decode the radiographic phenotype. *Cancer Res.* **2017**, *77*, e104–e107.
2. Zwanenburg, A.; Leger, S.; Vallières, M.; Löck, S. Image biomarker standardisation initiative. *J arXiv preprint arXiv:.07003* **2016**.
3. Haralick, R.M.; Shanmugam, K.; Dinstein, I.H.J.I.T.o.s., man,; cybernetics. Textural features for image classification. **1973**, 610–621.
4. Sun, C.; Wee, W.G. Neighboring gray level dependence matrix for texture classification. *Computer Vision, Graphics, and Image Processing* **1983**, *23*, 341–352.
5. Galloway, M. Texture classification using gray level run length. *J. Computer Graphics Image, Processing.* **1975**, *4*, 172–179.
6. Chu, A.; Sehgal, C.M.; Greenleaf, J.F. Use of gray value distribution of run lengths for texture analysis. *J. Pattern Recognition Letters* **1990**, *11*, 415–419.
7. Xu, D.-H.; Kurani, A.S.; Furst, J.D.; Raicu, D.S. Run-length encoding for volumetric texture. *J Heart* **2004**, *27*, 452–458.
8. Tang, X. Texture information in run-length matrices. *J IEEE transactions on image processing,* **1998**, *7*; 1602–1609
9. Thibault, G.; Fertil, B.; Navarro, C.; Pereira, S.; Cau, P.; Levy, N.; Sequeira, J.; Mari, J.-L. Shape and texture indexes application to cell nuclei classification. *International Journal of Pattern Recognition Artificial Intelligence* **2013**, *27*, 1357002.
10. Leek, J.T.; Johnson, W.E.; Parker, H.S.; Jaffe, A.E.; Storey, J.D. The sva package for removing batch effects and other unwanted variation in high-throughput experiments. *Bioinformatics* **2012**, *28*, 882–883, doi:10.1093/bioinformatics/bts034.
11. Gamer, M.; Lemon, J.; Fellows, I.; Singh, P.J.R.A.N.h.c.r.-p.o.w.p.i.i.p. Package irr: Various Coefficients of Interrater Reliability and Agreement (versión 0.84). **2012**.
12. Harrell Jr, F.E.; Harrell Jr, M.F.E. Package 'Hmisc'. *J CRAN* **2019**, 235–236.
13. Gautier, L.; Cope, L.; Bolstad, B.M.; Irizarry, R.A. affy—analysis of Affymetrix GeneChip data at the probe level. *Bioinformatics* **2004**, *20*, 307–315.
14. Smyth, G.K. Limma: linear models for microarray data. In *Bioinformatics and computational biology solutions using R and Bioconductor*, Springer: 2005; pp. 397–420.
15. Harrell Jr, F.E. rms: regression modeling strategies. R package version 5.1–2. *J Dept. Biostatist* **2017**.
16. Blanche, P. Package'timeROC': time-dependent ROC curve and AUC for censored survival data. *J Vienna: R Foundation for Statistical Computing* **2013**.
17. Therneau, T.M.; Lumley, T. Package 'survival'. *Survival analysis Published on CRAN* **2014**, *2*, 3.
